# A Rare Case of Cytomegalovirus in the Gut in an Immunocompetent Host With Ischemic Colitis

**DOI:** 10.7759/cureus.9432

**Published:** 2020-07-27

**Authors:** Balarama K Surapaneni, Shivani Priyadarshni, Vivek Choksi, Sufian Sorathia, Franklin Kasmin

**Affiliations:** 1 Internal Medicine, Aventura Hospital and Medical Center, Aventura, USA; 2 Gastroenterology, Aventura Hospital and Medical Center, Aventura, USA

**Keywords:** cmv colitis, ischemic colitis

## Abstract

Cytomegalovirus (CMV) infections are typically seen in individuals with immunosuppressive conditions such as malignancies, HIV/AIDS, and organ transplantation, and in patients on chemotherapy or steroids. Recurrent disease can occur if the virus reactivates due to disruption of immunity due to factors such as older age or immunosuppressive drugs. CMV is common, with a seroprevalence (CMV IgG-positive) of 40-100 % in adults, increasing with age. It has been reported that inflammatory bowel disease in remission can be exacerbated by CMV colitis or complicate steroids refractory colitis flare. For this reason, steroids should be cautiously started if clinical suspicion is high for CMV. We report a unique case of CMV colitis associated with severe ischemic colitis in an immunocompetent patient, with an excellent response to management with antiviral therapy.

## Introduction

Cytomegalovirus (CMV) infections are seen typically in individuals with immunosuppressive diseases such as malignancies, HIV/AIDS, and organ transplantation, and in patients on chemotherapy or steroids [1-4]. The first reported case of CMV colitis was published in 1992 [5]. The diagnosis for our patient was established after a rigorous diagnostic workup.

## Case presentation

A 68-year-old man with a medical history of pulmonary embolism and chronic obstructive pulmonary disease presented with chronic diarrhea of 8-10 weeks. His symptoms were associated with chills, lower abdominal discomfort, poor appetite, and weight loss of approximately 10-15 pounds. His physical examination was unremarkable. 

**Figure 1:**
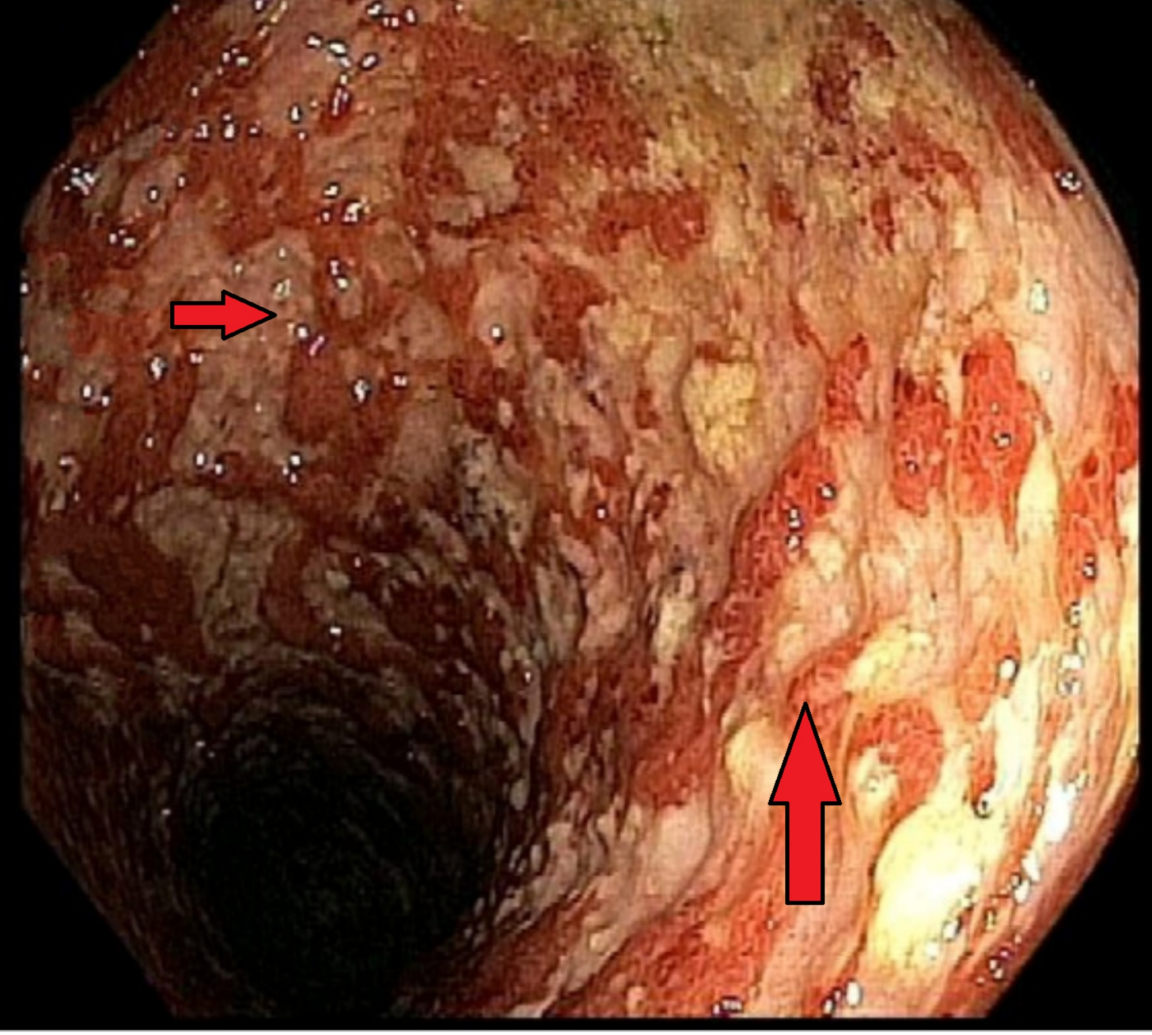
Colonoscopy Congestion (edema), erosions, erythema, friability, granularity, confluent ulcerations, deep ulcerations, and serpentine ulcerations can be seen in a continuous and circumferential pattern.

Laboratory data were significant for elevated erythrocyte sedimentation rate (ESR) and C-reactive protein (CRP). Stool studies were negative for *Cryptosporidium*, Giardia, *Campylobacter*, *Shigella*, *Clostridioides difficile*, ova, and parasites. CT scan of the abdomen noted findings suggestive of inflammatory bowel disease (IBD) with diffuse areas of active inflammation in the colon. Colonoscopy the following day reported inflammation characterized by congestion (edema), erosions, erythema, friability, granularity, confluent ulcerations, deep ulcerations, and serpentine ulcerations in a continuous and circumferential pattern from the sigmoid colon to the terminal ileum, sparing the mid-sigmoid colon, distal sigmoid colon, and the rectum (Figure 1). The patient was started on steroid and mesalamine therapy. Slowly, the patient started improving clinically, tolerating diet, and participating in physical therapy. He was discharged with recommendations for close follow-up with the gastroenterology service.

However, the following week, he was re-admitted with worsening symptoms. On admission, he was showing signs of sepsis (febrile, hypotensive, and tachycardic) with lactic acidosis. A repeat CT scan revealed worsening diffuse colitis. *Clostridioides difficile* was re-checked and was negative. He was admitted with “severe IBD exacerbation,” with failure to respond to outpatient therapy. He was started on intravenous steroids, antibiotics, and hydration. He had a moderate improvement over the next 48 to 72 hours. He was later discharged on oral steroid and mesalamine therapy.

The week after, he was admitted for the third time with further worsening of symptoms. He had lost approximately 25 pounds in the last two to three weeks. He was again re-admitted with “severe IBD exacerbation,” with failure to respond to outpatient therapy. The patient underwent repeat stool studies including *Clostridioides difficile*, which were negative. He was started on intravenous steroids, antibiotics, and hydration. His symptoms persisted despite aggressive treatment. Due to his anorexia and severe malnutrition, he was started on total parenteral nutrition (TPN), and biologic therapy was initiated. However, his symptoms continued to worsen. Colorectal surgery service was consulted, and he underwent open subtotal colectomy with splenic flexure mobilization, end ileostomy, and distal descending colon mucous fistula. Histopathology slides from the surgical specimen were concerning for ischemic bowel rather than IBD.

**Figure 2:**
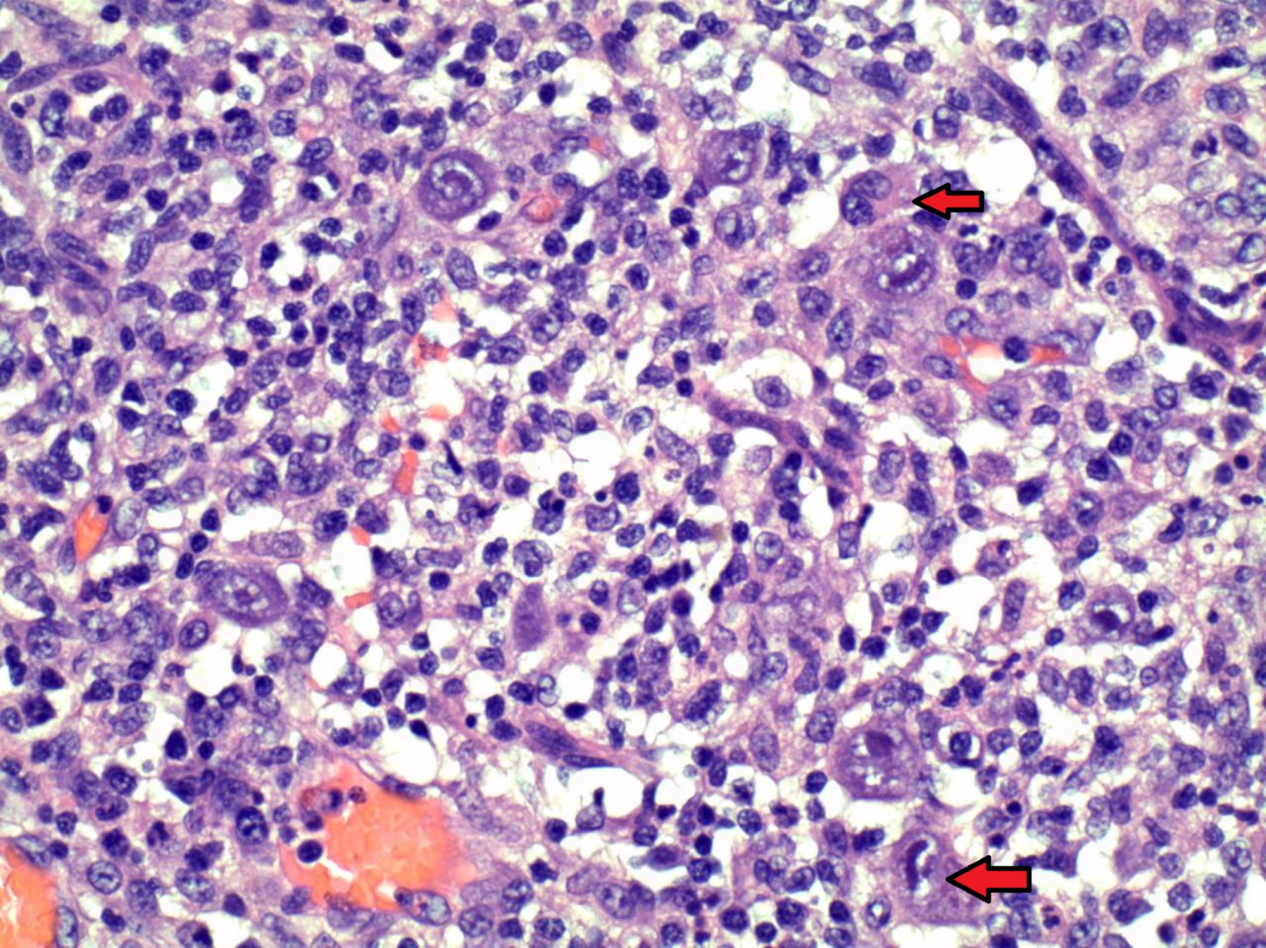
CMV inclusion bodies Photomicrograph of hematoxylin and eosin stained slide at high power showing owl’s eye inclusion bodies on histology from surgical specimen at magnifications of ×400. CSF, cerebrospinal fluid

Unfortunately, despite all the interventions, the patient was not really improving clinically; therefore, he was taken for emergent exploratory laparotomy. Laparotomy revealed diffusely ischemic and distended small bowel from right below the fascia at the level of end ileostomy to approximately 30 cm from the ligament of Treitz. Pathology was not consistent with IBD. It instead revealed ischemic ileocolitis with superimposed CMV colitis with the presence of CMV inclusion bodies (Figure 2). Serology was noted for CMV IgM antibodies indicating an acute infection. IV ganciclovir was initiated, and steroids were discontinued. Despite aggressive clinical manifestations, he dramatically improved over a few days. His diet was advanced and TPN was discontinued. He was eventually discharged on PO valganciclovir.

## Discussion

CMV causes a primary infection followed by the establishment of a latent period. Recurrent disease can occur if the virus reactivates due to disruption of immunity due to factors such as older age or immunosuppressive drugs. CMV is common, with a seroprevalence (CMV IgG-positive) of 40-100% in adults, increasing with age [6]. 

In a recent study in which 44 immunocompetent patients had been diagnosed with CMV colitis, it was noteworthy that only 10 of those patients had no associated co-morbid conditions. However, remaining of the 34 patients had various co-morbid conditions impairing the host defense function (pregnancy, renal disease, diabetes, malignancy) [7]. Interestingly, age over 55 years was related to a poor end result. These findings were similar to our patient, who also had advanced age with multiple co-morbidities.

Endoscopy and biopsy are necessary when suspicion of CMV colitis is present. Histology slides are noted for owl’s eye inclusion bodies, which are specific for supporting the diagnosis of CMV. However, histology has low sensitivity and can miss infections. Therefore, immunohistochemistry or simple hematoxylin and eosin staining should be used to improve sensitivity if an index of suspicion of CMV colitis remains high. There is a clear relationship between the detection of owl's eye inclusion bodies and the polymerase chain reaction (PCR) detection of CMV in the gut [8].

According to scientific literature, IBD in remission [9] can be exacerbated by CMV colitis or complicated by steroids leading to refractory colitis flare [10]. Steroids should be cautiously started if clinical suspicion is high for CMV. 

In our case, the patient endoscopic findings imitated IBD and ischemic colitis. The implementation of multiple unsuccessful therapeutic strategies forced us to explore further etiologies. Similar findings were reported in a retrospective study of 12 patients and suggested that an early diagnosis of CMV colitis has a favorable outcome on prognosis [11].

The explanation of CMV colitis can be postulated to two mechanisms: primary and secondary. The primary mechanism is CMV, which is responsible for proliferation and inflammation in endothelial cells, predisposing to vasculitis and ulceration, and, finally, leading to ischemic colitis [12]. The secondary mechanism is due to prior conditions such as IBD or ischemic colitis, which can lead to mucosal injury causing local immune suppression. Such situations may allow CMV infection to co-exist with IBD or ischemic colitis. In our patient, we noticed that severe ischemic colitis preceded the CMV infection as the endoscopic biopsy was initially negative. However, subsequent surgical specimen detected CMV inclusion bodies. The prognosis of CMV colitis is good if diagnosed and treated early.

With this case presentation, we hope to increase awareness about CMV colitis in the medical community and to consider the diagnosis even in immunocompetent patients once the other common etiologies are ruled out.

## Conclusions

This case illustrates the severity of this rare but life-threatening disease. CMV colitis is usually seen in individuals with immunosuppression. However, our patient was healthy prior to illness and had an atypical presentation of CMV colitis requiring rigorous workup. The purpose of this case report is to increase awareness about this clinical condition among medical professionals.
